# Impacts of Neighborhood Persistent Poverty and Socioeconomic Status on Hepatocellular Carcinoma Outcomes: A Large Population‐Based Cohort Study

**DOI:** 10.1002/cam4.71721

**Published:** 2026-03-17

**Authors:** Mohamed I. Elsaid, Holli A. Loomans‐Kropp, Yesung Kweon, Vinod K. Rustgi, Cecilia Dapino, Jesse J. Plascak, Samilia Obeng‐Gyasi, Chyke Doubeni, Na Li, Khalid Mumtaz, Electra D. Paskett

**Affiliations:** ^1^ Division of Biostatistics and Population Health, Department of Biomedical Informatics College of Medicine, The Ohio State University Columbus Ohio USA; ^2^ Division of Medical Oncology, Department of Internal Medicine College of Medicine, the Ohio State University Columbus Ohio USA; ^3^ Center for Biostatistics The Ohio State University Columbus Ohio USA; ^4^ Cancer Control The Ohio State University Comprehensive Cancer Center Columbus Ohio USA; ^5^ Division of Cancer Prevention Control, Department of Internal Medicine College of Medicine, the Ohio State University Columbus Ohio USA; ^6^ Division of Gastroenterology and Hepatology Rutgers Robert Wood Johnson Medical School Piscataway New Jersey USA; ^7^ Center for Liver Diseases and Masses Rutgers Robert Wood Johnson Medical School Piscataway New Jersey USA; ^8^ Case Western Reserve University Cleveland Ohio USA; ^9^ Division of Surgical Oncology, Department of Surgery The Ohio State University Wexner Medical Center and James Cancer Hospital Columbus Ohio USA; ^10^ Department of Family and Community Medicine The Ohio State University Wexner Medical Center Columbus Ohio USA; ^11^ Division of Gastroenterology, Hepatology, and Nutrition, Department of Internal Medicine College of Medicine, the Ohio State University Columbus Ohio USA

**Keywords:** causal inference, health disparities, liver cancer, social determinants of health, survival analysis

## Abstract

**Background:**

Hepatocellular carcinoma (HCC) survival in the United States varies sharply by neighborhood disadvantage.

**Aim:**

To determine whether residence in persistently impoverished or low‐SES census tracts is independently associated with lower all‐cause and HCC‐specific survival.

**Methods:**

We identified 51,323 adults with HCC using a population‐based retrospective cohort from the Surveillance, Epidemiology, and End Results Research Plus Specialized Database (2006–2020). Two census tract‐level socioeconomic exposures were defined: persistent poverty (≥ 20% living below the poverty line for approximately 30 years) and low SES (Yost Index first quintile). Overlap Propensity Score Weighting, combined with marginal structural models, estimated the 1‐, 5‐, 10‐, and 15‐year risks of all‐cause and HCC‐specific mortality.

**Results:**

The median follow‐up was 16 months, 6058 (11.8%) lived in persistently impoverished tracts, and 9863 (19.5%) lived in low‐SES tracts. After weighting, residents of persistently impoverished areas had a 1‐year all‐cause mortality risk of 46.0% vs. 40.3% (RD, 5.6%; 95% CI, 4.4% to 6.9%; RR, 1.14; 95% CI, 1.11 to 1.17) and an HCC‐specific mortality risk of 33.3% vs. 28.6% (RD, 4.8%; 95% CI, 3.2% to 6.3%; RR, 1.17; 95% CI, 1.11 to 1.22). Living in low‐SES tracts raised 1‐year all‐cause mortality risk to 32.5% vs. 30.1% (RD, 4.8%; 95% CI, 3.6% to 6.0%; RR, 1.12; 95% CI, 1.09 to 1.15) and HCC‐specific mortality risk to 32.5% vs. 30.1% (RD, 2.5%; 95% CI, 1.4% to 3.5%; RR, 1.08; 95% CI, 1.05 to 1.12).

**Conclusions:**

Both persistent neighborhood poverty and contemporary low SES independently contribute to significant increases in mortality risk after HCC diagnosis.

**Abbreviations**
ACS: American Community Survey
AFP: Alpha‐Fetoprotein
AJCC: American Joint Committee on Cancer
DAG: Directed Acyclic Graph
HCC: Hepatocellular Carcinoma
ICD‐O‐3: The International Classification of Diseases for Oncology
IRD: Incidence Rate Difference
OPSW: Overlap Propensity Score Weighting
RD: Risk Difference
RR: Risk Ratio
SD: Standard Deviation
SEER: Surveillance, Epidemiology, and End Results
SES: Socioeconomic Status

## 1 Introduction

Despite extensive clinical and public health efforts, Hepatocellular Carcinoma (HCC) incidence in the United States (US) has tripled since 1980, and five‐year survival remains only about 22%, making it the fifth and seventh leading cause of cancer‐related death among men and women, respectively [1, 2]. Socioenvironmental stressors significantly impact HCC outcomes along the cancer control continuum, with disparities evident across racial, ethnic, and socioeconomic lines [3, 4, 5, 6, 7, 8, 9, 10]. These disparities are rooted in multifaceted factors, including socioeconomic factors, sociocultural environments, neighborhood characteristics, healthcare access, quality of care, and discrimination, all of which contribute to disparities in HCC surveillance, treatment access, and outcomes [11, 12]. Limited access to healthcare, cultural barriers, systemic inequities, and stigma further exacerbate these disparities, especially for minoritized groups [13].

Area‐level socioeconomic deprivation is a marker for cumulative social and environmental disadvantages that affect cancer risk, stage at diagnosis, treatment access, and survival [14, 15, 16, 17, 18, 19, 20]. However, most HCC studies have treated deprivation as a single static construct, typically measured with composite indices derived from a single measure of socioeconomic deprivation [14, 15, 16, 17, 18, 19, 20, 21]. This approach conflates two related yet distinct dimensions: (i) persistent poverty, chronic neighborhood‐level deprivation sustained over decades, and (ii) contemporaneous neighborhood socioeconomic status (SES). Failing to separate these constructs obscures how long‐standing structural inequities versus current resource deficits independently influence survival and limits the design of targeted interventions.

Using the Surveillance, Epidemiology, and End Results (SEER) Research Plus Specialized Data (2006–2020), which links individual cancer records to census‐tract measures of both persistent poverty and neighborhood SES, we conducted a population‐based cohort study to examine their independent associations with long‐term all‐cause and HCC‐specific survival. We hypothesized that residence in a persistently impoverished or low‐SES tract would each confer lower long‐term survival after rigorous adjustment for demographic, clinical, and treatment confounders.

## 2 Methods

### 2.1 Study Design and Data Source

We performed a population‐based retrospective cohort study using the SEER Research Plus Specialized Database (17 registries, 2006–2020), which links individual cancer records to census‐tract attributes, including rurality, persistent poverty, and SES. The dataset captures patient demographics, tumor characteristics, initial treatment, and vital status for approximately 48% of the U.S. population [22, 23]. This study was exempt from institutional review, as de‐identified, publicly available data is not considered human subjects research. We followed the STROBE guidelines for observational studies [24].

### 2.2 Study Population

**FIGURE 1:**
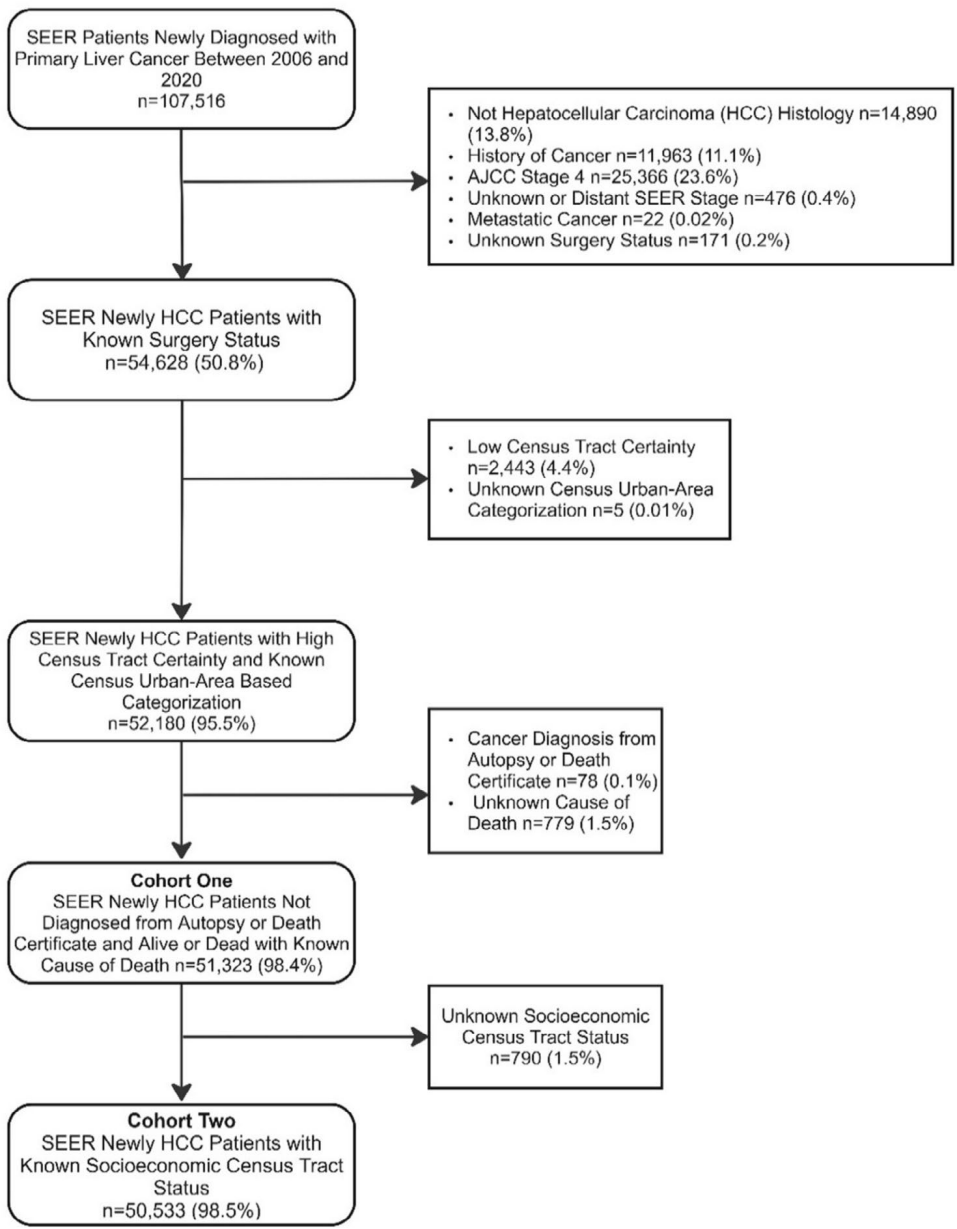
Study Schema.

The study cohort included newly diagnosed adult patients (18+) with primary liver cancer between 2006 and 2020. We restricted our cohort to HCC patients based on histology, as identified using the International Classification of Diseases for Oncology (ICD‐O‐3) codes 8170–8175 (Figure 1). We used two analytic cohorts based on exposure availability. Cohort one included all eligible patients and was used for analyses of persistent poverty. Cohort two applied the same eligibility criteria but additionally excluded patients with missing census‐tract SES because SES was required for those analyses. As such, we excluded cases with prior malignancy, stage IV/metastatic disease, autopsy/death‐certificate diagnosis, missing surgery status, low address certainty, or undefined urban‐area classification. After applying these eligibility criteria, we excluded 779 patients (1.5%) with an unknown cause of death (cohort one). Subsequently, we excluded an additional 790 patients (1.5%) with missing SES (cohort two).

### 2.3 Exposures

Two binary census‐tract–level exposures were evaluated: [1] persistent poverty, ≥ 20% of residents living below the federal poverty line for approximately 30 years, and [2] low SES, the first quintile of the validated Yost Index, which is based on seven tract‐level indicators: income, house value, rent, poverty, education, working‐class proportion, and unemployment [23]. Because SEER provides the Yost Index in national quintiles, we defined low SES a priori as the lowest quintile to capture concentrated disadvantage and to align the SES contrast with the binary persistent poverty exposure, while maintaining stable overlap for weighting and avoiding potential data sparsity. A detailed definition of exposure variables is included in the Method S1.

### 2.4 Outcome Assessment

Primary outcomes were the risksof all‐cause and HCC‐specific mortality ascertained from SEER standardized survival data. Follow‐up was from the first date of HCC diagnosis to (i) death for deceased patients or (ii) the date of last contact or the end of the study (December 31^st^, 2020) for patients presumed alive. For HCC‐specific death, deaths from causes other than HCC were treated as competing events, and patients were censored at the date of non‐HCC death (cause‐specific approach) [25]. Accordingly, HCC‐specific estimates should be interpreted as cause‐specific survival/risk under a competing‐risk framework rather than as cumulative incidence accounting for competing risks.

### 2.5 Variables of Interest

Detailed sociodemographic, clinical, and treatment variables were extracted from the SEER database. Racial and ethnic categories in SEER are social constructs reflecting case self‐identification [26]. The included pertinent variables are outlined in Table 1.

### 2.6 Approach to Confounding

We used Directed Acyclic Graphs (DAGs) to identify the minimal set of confounders necessary for unbiased total effect estimates. In cohort one, low SES census tract status was treated as a mediator of the relationship between persistent poverty and survival. Because persistent poverty and low SES are correlated but not interchangeable, we used DAG‐guided adjustment sets to estimate the total effect of persistent poverty and the association of low SES accounting for persistent poverty. Given that persistent poverty is a static, area‐level exposure measured over ~30 years, it precedes and is causally associated with more contemporaneous SES status. Consequently, we did not adjust for area‐level SES when estimating the total effect (i.e., both direct and indirect) of persistent poverty on survival. However, in cohort two, we considered persistent poverty a confounder of the relationship between area‐level SES and survival since it is associated with low SES, predisposes individuals to worse outcomes, and does not lie on the causal pathway from area‐level SES to death. This approach enabled us to isolate the distinct independent total effect of each exposure, persistent poverty, and low SES on HCC outcomes.

### 2.7 Statistical Analysis

A detailed statistical analysis is included in the Methods S2. We estimated propensity scores for each cohort via multivariable logistic regression (main effects + first‐order interactions). We then used each cohort's propensity scores to estimate Overlap Propensity Score Weights (OPSW) to account for confounding [27]. Overall and stratified characteristics were summarized using descriptive statistics, including means and Standard Deviations (SD) for continuous variables and frequencies and proportions for categorical variables. Differences between exposure groups were compared using Student's *t*‐tests or Wilcoxon Rank‐Sum tests for continuous variables, and χ^2^ or Fisher's exact tests for categorical variables. We used overlap‐weighted standardized mean differences to compare sociodemographic, clinical, and treatment variables according to exposure status. A standardized difference of less than 0.1 indicated negligible differences between patient characteristics and exposure status [28].

We fitted OPSW‐weighted marginal structural models using parametric pooled logistic regression, including an indicator for exposure, a flexible time‐varying intercept (with linear and quadratic terms), and interaction terms between exposure and time. The average 1‐, 5‐, 10‐, and 15‐year absolute risks, risk differences (RDs), and risk ratios (RRs) for all‐cause and HCC‐specific mortality were estimated using predicted values from the weighted marginal structural models. We used non‐parametric bootstrapping with 1000 replications to estimate 95% Confidence Intervals (CIs) for RDs and RRs. Two‐sided tests were considered statistically significant, with a significance level of 0.05. All analyses were performed using SAS version 9.4. and R 4.2.0.

### 2.8 Secondary and Sensitivity Analyses

We conducted secondary analyses using Kaplan–Meier and OPSW‐weighted Cox proportional hazards regression to enhance the comparability of our findings with other studies. In addition, we calculated incidence rates (IR) for each exposure group and the absolute Incidence Rate Differences (IRDs) of all‐cause and HCC‐specific mortalities. In a sensitivity analysis, we extended the study cohorts to include participants with stage IV/metastatic disease and repeated our OPSW‐weighted Cox analysis.

## 3 Results

### 3.1 Sample Characteristics

The study included 51,323 newly diagnosed patients with HCC in cohort one, of whom 50,533 met the inclusion criteria for cohort two (Figure 1). Table 1 shows the patient characteristics stratified by persistently impoverished residence status. An estimated 11.8% of patients lived in persistently impoverished areas. Compared with those not residing in persistently impoverished areas, patients living in persistently impoverished areas were younger (mean age 64.0 vs. 62.2; *p* < 0.001), more likely to be non‐Hispanic Black (9.8% vs. 31.7%; *p* < 0.001), Hispanic (20.1% vs. 30.6%; *p* < 0.001), single or never married (19.6% vs. 33.5%; *p* < 0.001), and living in urban census tracts (72.6% vs. 81.2%; *p* < 0.001). Overall, persistent poverty was associated with more advanced tumor characteristics, including higher proportions of regional SEER summary stage, AJCC stage III, and T3+ tumors (all P‐values < 0.05). Additionally, patients residing in persistently impoverished census tracts were less likely to receive surgical treatment (32.2% vs. 24.1%; *p* < 0.001), radiotherapy (11.2% vs. 9.6%; *p* < 0.001), and chemotherapy (42.1% vs. 38.7%; *p* < 0.001).

In cohort two, the prevalence of residence in low socioeconomic areas was 19.5% Table S1. Persistent poverty and low SES were correlated. Among patients residing in low‐SES tracts, 51.6% (5084/9863) also resided in persistently impoverished tracts, whereas only 1.8% (740/40,670) of those not residing in low‐SES tracts lived in persistently impoverished tracts Table S1. The associations between residence in low socioeconomic areas, demographics, clinical, and treatment characteristics mirrored those observed in persistently impoverished areas. After applying the OPSW, all characteristics were well‐balanced between those residing and those not living in persistently impoverished areas (Table 1). Similar results were observed when using OPSW to balance demographics, clinical, and treatment characteristics by residents of low socioeconomic areas status Table S1.

**TABLE 1:** Sociodemographic, clinical, and treatment characteristics of patients with hepatocellular carcinoma by residence in persistent persistently impoverished census tracts status, the surveillance, epidemiology, and end results (SEER) 2006–2020 (*n* = 51,323).

Patient characteristics	Unweighted			Weighted^a^		
	Not residing in persistently impoverished census tract	Residing in persistently impoverished census tract	Standardized difference^b^	Not residing in persistently impoverished census tract	Residing in persistently impoverished census tract	Standardized difference^d^
	*n* (%)	*n* (%)		%^c^	%^c^	
No. of Patients	45,265 (88.2)	6058 (11.8)		50.0	50.0	
**Demographics**						
Age, Years						
Mean (SD)	64.0 (10.5)	62.2 (9.8)	0.1767	62.6 (3.4)	62.5 (9.0)	0.0134
Median (25^th^, 75^th^)	63.0 (57.0, 71.0)	61.0 (56.0, 68.0)		62.0 (56.0, 68.0)	62.0 (56.0, 68.0)	
Age Group, Years						0^e^
20 to 50	3417 (7.6)	524 (8.7)	0.0404	8.7	8.7	
51 to 60	13,998 (30.9)	2230 (36.8)	0.1246	35.8	35.8	
61 to 70	16,300 (36.0)	2182 (36.0)	0.0002	35.9	35.9	
71+	11,550 (25.5)	1122 (18.5)	0.1694	19.7	19.7	
Sex						0^e^
Male	34,396 (76.0)	4586 (75.7)	0.0067	75.7	75.7	
Female	10,869 (24.0)	1472 (24.3)		24.3	24.3	
Race‐Ethnicity						0^e^
Non‐Hispanic White	23,119 (51.1)	1640 (27.1)	0.5075	30.7	30.7	
Non‐Hispanic Black	4431 (9.8)	1919 (31.7)	0.5607	26.4	26.4	
Non‐Hispanic American Indian/Alaska Native	416 (0.9)	61 (1.0)	0.0090	1.1	1.1	
Non‐Hispanic Asian or Pacific Islander	8072 (17.8)	581 (9.6)	0.2414	10.9	10.9	
Hispanic	9115 (20.1)	1851 (30.6)	0.2412	30.8	30.8	
Non‐Hispanic Unknown Race	112 (0.3)	6 (0.1)	0.0357	0.1	0.1	
Marital Status at Diagnosis						0^e^
Married or Partner	24,158 (53.4)	2216 (36.6)	0.3424	39.4	39.4	
Separated or Divorced	6191 (13.7)	1044 (17.2)	0.0985	16.9	16.9	
Single Never Married	8861 (19.6)	2030 (33.5)	0.3196	30.7	30.7	
Widowed	4077 (9.0)	514 (8.5)	0.0185	8.7	8.7	
Unknown	1978 (4.4)	254 (4.2)	0.0087	4.3	4.3	
Year of Diagnosis						0^e^
2006	1970 (4.4)	239 (4.0)	0.0204	4.0	4.0	
2007	2163 (4.8)	262 (4.3)	0.0218	4.4	4.4	
2008	2304 (5.1)	282 (4.7)	0.0202	4.7	4.7	
2009	2681 (5.9)	353 (5.8)	0.0041	5.8	5.8	
2010	2780 (6.1)	354 (5.8)	0.0126	5.9	5.9	
2011	2967 (6.6)	410 (6.8)	0.0085	6.7	6.7	
2012	3180 (7.0)	432 (7.1)	0.0041	7.1	7.1	
2013	3348 (7.4)	484 (8.0)	0.0223	7.9	7.9	
2014	3604 (8.0)	476 (7.9)	0.0039	7.8	7.8	
2015	3722 (8.2)	522 (8.6)	0.0142	8.6	8.6	
2016	3409 (7.5)	462 (7.6)	0.0036	7.7	7.7	
2017	3319 (7.3)	430 (7.1)	0.0091	7.2	7.2	
2018	3398 (7.5)	476 (7.9)	0.0132	7.7	7.7	
2019	3507 (7.8)	445 (7.4)	0.0152	7.5	7.5	
2020	2913 (6.4)	431 (7.1)	0.0270	7.0	7.0	
Census Urban‐Area Categorization						0^e^
All Urban	32,842 (72.6)	4920 (81.2)	0.2065	79.9	79.9	
Mostly Urban	7951 (17.6)	728 (12.0)	0.1568	12.8	12.8	
Mostly Rural	2519 (5.6)	169 (2.8)	0.1391	3.2	3.2	
All Rural	1953 (4.3)	241 (4.0)	0.0169	4.2	4.2	
**Clinical and Treatment Characteristics**						
Histological Type						0^e^
Hepatocellular carcinoma, NOS	44,853 (99.1)	5992 (98.9)	0.0180	98.9	98.9	
Hepatocellular carcinoma, Fibrolamellar	81 (0.2)	11 (0.2)	0.0006	0.2	0.2	
Hepatocellular carcinoma, Scirrhous	41 (0.1)	8 (0.1)	0.0124	0.1	0.1	
Hepatocellular carcinoma, Spindle Cell Variant	21 (0.1)	1 (0.02)	0.0169	0	0	
Hepatocellular carcinoma, Clear Cell Type	256 (0.6)	44 (0.7)	0.0201	0.7	0.7	
Hepatocellular carcinoma, Pleomorphic Type	13 (0.03)	2 (0.03)	0.0024	0.0	0.0	
SEER Summary Stage						0^e^
Localized	28,819 (63.7)	3778 (62.4)	0.0270	62.8	62.8	
Regional	16,446 (36.3)	2280 (37.6)		37.3	37.3	
AJCC Staging						0^e^
I	21,968 (48.5)	2910 (48.0)	0.0099	48.3	48.3	
II	11,308 (25.0)	1433 (23.7)	0.0309	24.0	24.0	
III	11,989 (26.5)	1715 (28.3)	0.0409	27.8	27.8	
Pathologic Grade						
Grade 1	4792 (10.6)	598 (9.9)	0.0236	10.0	10.0	0.0006
Grade 2	7081 (15.6)	847 (14.0)	0.0468	14.6	14.0	0.0178
Grade 3	2678 (5.9)	351 (5.8)	0.0052	5.9	5.8	0.0036
Grade 4	207 (0.5)	29 (0.5)	0.0031	0.4	0.5	0.0049
Unknown	30,507 (67.4)	4233 (69.9)	0.0534	69.0	69.7	0.0150
TNM‐T						0^e^
T0/T1	21,797 (48.2)	2896 (47.8)	0.007	48.0	48.0	
T2	11,260 (24.9)	1430 (23.6)	0.0297	23.9	23.9	
T3	7835 (17.3)	1161 (19.2)	0.0481	18.7	18.7	
T4	1542 (3.4)	202 (3.3)	0.0040	3.3	3.3	
Tx	2831 (6.3)	369 (6.1)	0.0068	6.1	6.1	
TNM‐N						0^e^
N0	43,356 (95.8)	5816 (96.0)	0.0112	96.0	96.0	
N1	1350 (3.0)	175 (2.9)	0.0056	2.9	2.9	
Nx	559 (1.2)	67 (1.1)	0.0120	1.1	1.1	
Surgery Status						
None	30,670 (67.8)	4595 (75.9)	0.1806	70.7	75.3	0.1028
Local tumor destruction	6520 (14.4)	688 (11.4)	0.0911	13.6	11.6	0.0600
Wedge or segmental resection	2861 (6.3)	294 (4.9)	0.0639	5.8	4.9	0.0366
Lobectomy	1607 (3.6)	149 (2.5)	0.0639	3.2	2.5	0.0397
Extended lobectomy	415 (0.9)	32 (0.5)	0.0459	0.8	0.5	0.0339
Hepatectomy	3038 (6.7)	287 (4.7)	0.0851	5.7	5	0.0331
Other Surgery	154 (0.3)	13 (0.2)	0.0239	0.3	0.2	0.0140
Radiotherapy						
Yes	5046 (11.2)	579 (9.6)	0.0522	10.5	9.6	0.0277
Refusal	184 (0.4)	33 (0.5)	0.0201	0.4	0.6	0.0234
No/Unknown^f^	40,035 (88.5)	5446 (89.9)	0.0467	89.1	89.8	0.0218
Chemotherapy						
Yes	19,060 (42.1)	2346 (38.7)	0.0690	41.9	38.7	0.0650
No/Unknown	26,205 (57.9)	3712 (61.3)		58.1	61.3	
Time From Diagnosis to Treatment, Months						
Mean (SD)	2.3 (2.3)	2.4 (2.4)	0.0508	2.4 (0.8)	2.4 (2.2)	0.0200
Median (25^th^, 75^th^)	2.0 (1.0, 3)	2.0 (1.0, 3.0)		2 (1, 3)	2 (1, 3)	
Time From Diagnosis to Treatment, Months						
1 month or less	14,018 (31.0)	1577 (26.0)	0.1095	29.1	26.2	0.0629
2 months	8027 (17.7)	876 (14.5)	0.0892	16.6	14.6	0.0548
3+ months	10,457 (23.1)	1314 (21.7)	0.0339	23.6	21.8	0.0419
Unknown	12,763 (28.2)	2291 (37.8)	0.2057	30.8	37.3	0.1387
AFP Pretreatment Interpretation						0^e^
Negative	9752 (21.5)	1083 (17.9)	0.0923	18.5	18.5	
Positive	20,163 (44.5)	2947 (48.7)	0.0823	48.0	48.0	
Unknown^g^	15,350 (33.9)	2028 (33.5)	0.0092	33.5	33.5	
Tumor Size, mm						
Mean (SD)	53.0 (49.2)	55.5 (47.0)	0.0504	54.8 (16.9)	55.0 (42.3)	0.0043
Median (25^th^, 75^th^)	40 (25, 67)	42 (26, 71)		41 (25, 70)	42 (26, 70)	
Tumor Size Group, mm						0^e^
< 50	25,875 (57.2)	3206 (52.9)	0.0853	53.8	53.8	
≥ 50	16,441 (36.3)	2361 (39.0)	0.0547	38.4	38.4	
Unknown	2949 (6.5)	491 (8.1)	0.0611	7.8	7.8	
Total Number of in situ/malignant Tumors						
Mean (SD)	1.1 (0.3)	1.1 (0.2)	0.0743	1.1 (0.1)	1.1 (0.2)	0.0010
Median (25^th^, 75^th^)	1.0 (1.0, 1.0)	1.0 (1.0, 1.0)		1.0 (1.0, 1.0)	1.0 (1.0, 1.0)	
Total Number of in situ/malignant Tumors						0^e^
1	42,391 (93.7)	5777 (95.4)	0.0751	95.2	95.2	
2+	2874 (6.4)	281 (4.6)		4.8	4.8	

Abbreviations: NOS, not otherwise specified; SD: standard deviation.

^a^ Using overlap weighting. The weighting aims to construct a pseudo‐sample in which persistent poverty status is independent of the baseline demographics and cancer characteristics influencing the likelihood of residing in a persistent poverty census tract.

^b^ Absolute difference in means or proportions divided by pooled standard deviation. The imbalance between the persistent poverty census tract and non‐persistent poverty census tract groups is defined as an absolute value greater than 0.10; smaller values indicate better balance.

^c^ Overlap weighted proportions.

^d^ Overlap‐weighted standardized differences. All patient baseline demographics and cancer characteristics were used to estimate the weights.

^e^ Overlapping weighting resulted in an exact balance for this variable.

^f^ Including recommended and unknown if administered.

^g^ Including test ordered, results not in the chart, information not collected, not documented, not assessed, or unknown if assessed.

### 3.2 Effects of Living in Persistently Impoverished Areas on Survival

**FIGURE 2:**
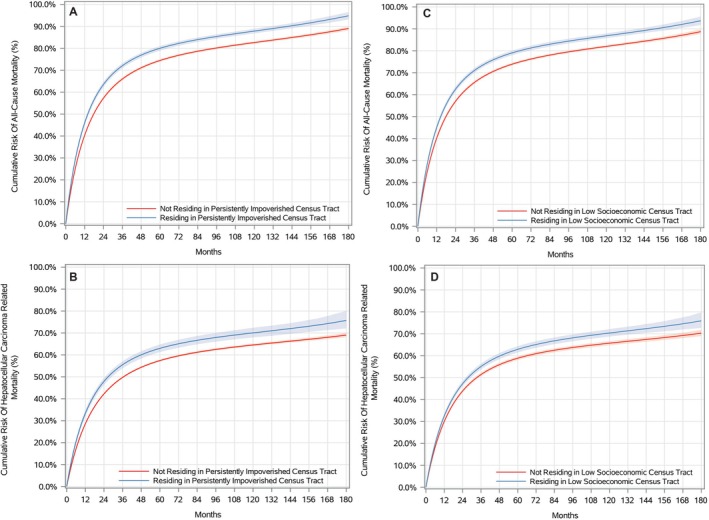
Adjusted 1‐, 5‐, 10‐, and 15‐year (A) All‐Cause and (B) HCC‐Specific Mortality Risks for Participants with HCC Residing in Persistently Impoverished Census Tracts, Compared to Those Not Residing in Persistently Impoverished Census Tracts (*N* = 51,323) and Adjusted 1‐, 5‐, 10‐, and 15‐year (C) All‐Cause and (D) HCC‐specific Mortality Risks for Participants with HCC Residing Versus Not Residing in a Low Socioeconomic Census Tract (*n* = 50,533).

The median (interquartile range) follow‐up time for all patients was 16.0 (5.0–40.0) months, with 12.0 (4.0–32.0) months and 17.0 (5.0–41.0) months for those who resided and did not reside in persistently impoverished areas, respectively. During the 15 years of follow‐up, residents of persistently impoverished areas accounted for 4589/6058 (75.8%) deaths from all causes, whereas non‐residents accounted for 31,080/45,265 (68.7%). Significantly increased risk of all‐cause mortality was observed at 1‐, 5‐, 10‐, and 15‐year intervals for individuals living in persistently impoverished areas (Figure 2A and Table 2). The OPSW‐adjusted 15‐year all‐cause mortality risks were 94.8% (93.2% to 96.5%) for those living and 89.1% (95% CI: 88.2% to 89.9%) for patients not residing in persistently impoverished areas (adjusted RD = 5.8%, 95% CI: 3.9% to 7.5%; adjusted RR = 1.06, 95% CI: 1.04 to 1.08).

**TABLE 2:** Estimated 15‐year overlapping weight adjusted risks^a^ of all‐cause and hepatocellular carcinoma specific mortalities comparing patients residing vs. not residing in a persistently impoverished census tract, patients with hepatocellular carcinoma, the surveillance, epidemiology, and end results (SEER) 2006–2020 (*n* = 51,323).

Follow‐Up (Years)	Adjusted cumulative risk (%) (95% CI)		Adjusted risk difference^b^ (%) (95% CI)	Adjusted risk ratio^c^ (95% CI)
	Residing in persistently impoverished census tract	Not residing in persistently impoverished census tract		
All‐cause mortality risk				
1	46.0 (44.6 to 47.2)	40.3 (39.6 to 41.0)	5.6 (4.4 to 6.9)	1.14 (1.11 to 1.17)
5	80.0 (78.9 to 81.0)	74.4 (73.8 to 75.0)	5.6 (4.5 to 6.6)	1.07 (1.06 to 1.09)
10	87.8 (86.8 to 88.9)	82.6 (82.0 to 83.2)	5.2 (4.1 to 6.4)	1.06 (1.05 to 1.08)
15	94.8 (93.2 to 96.5)	89.1 (88.2 to 89.9)	5.8 (3.9 to 7.5)	1.06 (1.04 to 1.08)
Hepatocellular carcinoma specific mortality risk				
1	33.3 (31.8 to 34.8)	28.6 (28.1 to 29.0)	4.8 (3.2 to 6.3)	1.17 (1.11 to 1.22)
5	63.0 (61.3 to 64.7)	57.4 (56.9 to 57.9)	5.6 (3.7 to 7.4)	1.10 (1.07 to 1.13)
10	70.0 (68.0 to 72.4)	64.5 (63.9 to 65.2)	5.5 (3.3 to 7.9)	1.09 (1.05 to 1.12)
15	75.7 (72.1 to 80.1)	69.0 (68.1 to 70.0)	6.7 (2.9 to 11.4)	1.10 (1.04 to 1.17)

Abbreviation: CI, confidence interval.

^a^ Adjusted using overlapping weights estimated using sex, age, race‐ethnicity, marital status at diagnosis, year of diagnosis, census urban‐area categorization, hepatocellular carcinoma histological type, SEER summary stage, AJCC staging, TNM‐N, TNM‐t, AFP pretreatment interpretation, tumor size, and total number of in situ/malignant tumors.

^b^ Differences between residing and not residing in a persistently impoverished census tract.

^c^ Comparing those residing vs. not residing in a persistently impoverished census tract.

A parallel pattern emerged for HCC‐specific death in cause‐specific analyses (censoring non‐HCC deaths). In the HCC‐specific analysis, 2995/6058 (48.8%) and 20,744/45,265 (45.8%) patients died of HCC in the persistently and non‐persistently impoverished groups, respectively. The 15‐year risk of HCC‐specific mortality was significantly higher in residents of persistently impoverished areas 75.7% (95% CI: 72.1% to 80.1%) vs. those not residing in persistently impoverished 69.0% (95% CI: 68.1% to 70.0%) areas (adjusted RD = 6.7%, 95% CI: 2.9% to 11.4%; adjusted RR = 1.10, 95% CI: 1.04 to 1.17) (Figure 2B and Table 2).

### 3.3 Effects of Living in Low Socioeconomic Areas on Survival

In cohort two, the median (interquartile range) follow‐up time for all patients was 16.0 (5.0–40.0) months, with 13.0 (4.0–32.0) months and 17.0 (5.0–42.0) months for those who resided and did not reside in a low socioeconomic census tract, respectively. During the 15 years of follow‐up, residents of low socioeconomic census tracts experienced 7464/9863 (75.7%) deaths due to all causes, whereas those not residing in low socioeconomic census tracts experienced 27,684/40,670 (68.1%) deaths. The OPSW‐adjusted 15‐year all‐cause mortality risk estimates were 93.7% (95% CI: 91.8% to 95.6%) for individuals residing in low socioeconomic census tracts and 88.7% (95% CI: 87.7% to 89.8%) for those not living in low socioeconomic census tracts (adjusted RD = 5.0%, 95% CI: 2.8% to 7.0%; adjusted RR = 1.06, 95% CI: 1.03 to 1.08) (Figure 2C and Table 3).

**TABLE 3:** Estimated 15‐year overlapping weight adjusted risks^a^ of all‐cause and hepatocellular carcinoma specific mortalities comparing patients residing vs. not residing in low socioeconomic census tract, patients with hepatocellular carcinoma, the surveillance, epidemiology, and end results (SEER) 2006–2020 (*n* = 50,533).

Follow‐Up (Years)	Risk of mortality (%) (95% CI)		Risk difference (%) (95% CI)^b^	Risk ratio (95% CI)^c^
	Residing in low socioeconomic census tract	Not residing in low socioeconomic census tract		
All‐cause mortality risk				
1	45.0 (43.9 to 46.2)	40.2 (39.5 to 41.0)	4.8 (3.6 to 6.0)	1.12 (1.09 to 1.15)
5	79.0 (78.0 to 80.1)	73.9 (73.3 to 74.5)	5.2 (4.1 to 6.3)	1.07 (1.06 to 1.08)
10	86.8 (85.8 to 87.9)	82.0 (81.4 to 82.6)	4.8 (3.7 to 6.1)	1.06 (1.04 to 1.08)
15	93.7 (91.8 to 95.6)	88.7 (87.7 to 89.8)	5.0 (2.8 to 7.0)	1.06 (1.03 to 1.08)
Hepatocellular carcinoma specific mortality risk				
1	32.5 (31.5 to 33.7)	30.1 (29.4 to 30.8)	2.5 (1.4 to 3.5)	1.08 (1.05 to 1.12)
5	62.9 (61.5 to 64.3)	58.9 (58.0 to 59.7)	4.0 (2.6 to 5.6)	1.07 (1.04 to 1.10)
10	70.4 (68.7 to 72.1)	65.7 (64.8 to 66.6)	4.6 (3.0 to 6.6)	1.07 (1.05 to 1.10)
15	75.9 (72.7 to 79.8)	70.3 (68.9 to 71.8)	5.6 (2.3 to 9.6)	1.08 (1.03 to 1.14)

Abbreviation: CI, confidence interval.

^a^ Adjusted using overlapping weights estimated using sex, age, race‐ethnicity, marital status at diagnosis, year of diagnosis, census urban‐area categorization, hepatocellular carcinoma histological type, SEER summary stage, AJCC staging, TNM‐N, TNM‐t, AFP pretreatment interpretation, tumor size, total number of in situ/malignant tumors, and residing in persistently impoverished census tract.

^b^ Differences between residing and not residing in a low socioeconomic census tract.

^c^ Comparing those residing vs. not residing in a low socioeconomic census tract.

In cause‐specific analyses of HCC‐specific death (censoring non‐HCC deaths), 4840/9863 (49.1%) and 18,560/40,670 (45.6%) patients succumbed to HCC in low‐ and not low‐socioeconomic census tracts, respectively. The 15‐year HCC‐specific mortality risk was significantly higher among residents of low socioeconomic census tracts at 75.9% (95% CI: 72.7% to 79.8%) compared to those not residing in low socioeconomic census tracts at 70.3% (95% CI: 68.9% to 71.8%) (adjusted RD = 5.6%, 95% CI: 2.3% to 9.6%; adjusted RR = 1.08, 95% CI: 1.03 to 1.14) (Figure 2D and Table 3).

### 3.4 Secondary Analysis

In the Kaplan–Meier analysis, residing in persistently impoverished areas was associated with increased all‐cause and HCC‐specific mortality risks Figures S1A–B. Over 15 years, all‐cause mortality IRs were 37.8 (95% CI: 36.7 to 38.9) vs. 27.0 (95% CI: 26.7 to 27.3); IRD was 10.8 (95% CI: 9.7 to 12.0) deaths per 100 person‐years. OPSW‐adjusted Cox models showed a Hazard Ratio (HR) of 1.19 (95% CI: 1.15 to 1.22) for all‐cause and HR 1.15 (95% CI: 1.11 to 1.20) for HCC‐specific mortality hazards. Residents of low‐socioeconomic areas showed similar patterns Figures S1C–D. All‐cause mortality risk was 37.6 (95% CI: 36.7 to 38.4) vs. 26.2 (95% CI: 25.9 to 26.5); IRD was 11.4 (95% CI: 10.5 to 12.3) deaths per 100 person‐years. Adjusted HR was 1.16 (95% CI: 1.13 to 1.20) for all‐cause mortality and HR 1.11 (95% CI: 1.07 to 1.16) for HCC‐specific mortality Table S2.

### 3.5 Sensitivity Analysis

Our sensitivity analyses on the extended cohorts to stage IV/metastatic disease confirmed the robustness of our main findings. Residents of persistently impoverished areas had a 15% higher OPSW‐adjusted all‐cause mortality hazard (HR = 1.15, 95% CI: 1.12 to 1.18) and an 11% higher HCC‐specific OPSW‐adjusted mortality hazard (HR = 1.11, 95% CI: 1.08 to 1.15) compared to their non‐persistently impoverished area counterparts. Similarly, residence in low socioeconomic areas was associated with a 13% higher OPSW‐adjusted all‐cause mortality hazard (HR = 1.13, 95% CI: 1.10 to 1.16) and an 8% increased OPSW‐adjusted HCC‐specific mortality hazard (HR = 1.08, 95% CI: 1.05 to 1.12) than those who did not reside in low socioeconomic areas.

## 4 Discussion

In this large, population‐based analysis employing a robust causal inference framework, we found that both residing in a persistently impoverished and living in a low socioeconomic census tract were independently associated with higher all‐cause and HCC‐specific mortality risks. Secondary and sensitivity analyses confirmed our main findings, underscoring the consistent impact of structural disadvantages on long‐term survival outcomes. Persistent poverty and contemporaneous neighborhood SES represent related but distinct dimensions of disadvantage. Persistent poverty reflects sustained poverty over decades and may capture long‐term structural disinvestment that shapes access to prevention, early detection, and cancer care infrastructure. In contrast, contemporaneous SES reflects neighborhood resources at the time of diagnosis. In our causal framework, we estimated the total effect of persistent poverty (unconditioned on downstream SES) and, separately, the association between low SES and persistent poverty. Together, these findings support interventions that address both entrenched structural deprivation and present‐day resource barriers affecting HCC detection and treatment.

Our findings align with a growing body of literature suggesting that area‐level socioeconomic disadvantages critically shape HCC outcomes [14, 15, 16, 17, 18, 19, 20]. Prior studies have reported elevated mortality rates in persistently impoverished counties, with liver and intrahepatic bile duct cancer‐related mortality noted to be 26% higher in these communities compared to non‐persistently impoverished areas [17, 18]. Similarly, an investigation of 5962 HCC patients in Ohio found that individuals residing in the most disadvantaged neighborhoods faced a 15% higher hazard of death within 5 years of diagnosis [29], reflecting the pervasive influence of neighborhood‐level disparities on cancer survival. Moreover, residing in high‐deprivation neighborhoods was identified as an independent risk factor for worse survival in early‐stage HCC [19]. By employing a rigorous causal inference framework—with OPSW, a multi‐decade measure of area‐level persistent poverty and socioeconomic status, and granular census tract data—our study elucidates the interplay between chronic and current socioeconomic disadvantages, demonstrating how both exposures independently produce significant, long‐term survival disparities among patients with HCC.

Disparities in HCC outcomes are not only shaped by chronic poverty and residence in low socioeconomic areas but also by intersecting factors such as race, ethnicity, and other social factors [7, 9]. Studies consistently indicate that Black and Hispanic patients with HCC are significantly less likely to receive potentially curative treatments than their white counterparts, often presenting with advanced disease and poorer survival [9]. Similarly, lower SES, characterized by limited income and educational opportunities, further amplifies this burden, with uninsured and publicly insured individuals showing delayed or no treatment [7]. Patients residing in persistently impoverished or low‐SES tracts were more likely to be non‐Hispanic Black or Hispanic and were also more likely to present with advanced tumor characteristics and less likely to receive surgery, radiotherapy, or chemotherapy. This observation is echoed in prior work suggesting that high‐deprivation neighborhoods exhibit markedly lower rates of surgical intervention for early‐stage HCC, potentially driven by higher comorbidity burdens [19]. Taken together, our results highlight how persistent poverty and residence in low socioeconomic census tracts each independently exacerbate long‐term mortality risks, underscoring the pressing need to address both the immediate resource deficits and broader structural inequalities that perpetuate disparities in liver cancer care.

Low adherence to HCC surveillance recommendations is a key factor potentially driving the higher rates of advanced HCC observed in residents of impoverished and low socioeconomic areas. Current estimates place overall HCC surveillance rates between 18% and 30% [30, 31, 32]. Race, ethnicity, and other social factors further compound this poor surveillance compliance [32, 33, 34]. Minoritized ethnicity is a key predictor of screening, with variations observed across different ethnic groups [33, 35]. These patterns are exemplified by data from Ohio, where residents of low‐opportunity neighborhoods are 64% less likely to adhere to surveillance guidelines than those in higher‐opportunity areas [21]. Consequently, nearly half of all newly diagnosed cases present at an advanced stage, an estimated 44% of newly diagnosed HCC are in the regional or distant stages [36], and only about one‐third are detected through surveillance [37, 38, 39, 40, 41, 42]. Consistent with these findings, our analysis revealed a higher proportion of advanced‐stage HCC among patients in persistently impoverished and low socioeconomic census tracts, underscoring how inadequate surveillance may drive delayed detection in these communities. Moreover, structural barriers such as inadequate literacy, mental health challenges, and complexities within the healthcare system can undermine patients' ability to engage in consistent HCC surveillance, perpetuating advanced‐stage disease at diagnosis and subsequent poorer survival outcomes [6].

### 4.1 Strengths and Limitations

This investigation has several strengths, including a large, population‐based sample drawn from SEER's Research Plus Specialized Database, which captures nearly half of the US population. Leveraging advanced causal inference methods, such as OPSW and marginal structural models, enabled robust confounder control and more accurate effect estimations. Additionally, the granular measurement of area‐level persistent poverty and low SES provides a nuanced understanding of how chronic versus current disadvantage independently shapes long‐term survival. Importantly, our secondary and sensitivity analyses, including extended cohorts with metastatic disease and supplementary Cox proportional hazards models, confirmed the robustness of the observed associations, reinforcing the reliability of our findings.

Nevertheless, potential limitations remain. Despite rigorous adjustments, residual confounding may persist due to unmeasured factors (e.g., individual‐level socioeconomic status, comorbidities, genetic predispositions, Child‐Pugh or MELD scores) unavailable in SEER. The retrospective nature of our study also precludes establishing a definitive temporal sequence for some confounders. This study included patients diagnosed in 2020, when COVID‐19 may have disrupted cancer care and increased competing mortality. Because follow‐up ended on December 31, 2020, 2020 diagnoses contributed limited follow‐up, and any pandemic‐related effects would most likely influence early follow‐up estimates. Although exclusions for unknown cause of death and missing SES were small (1.5% each), selection bias is possible if missingness was associated with both neighborhood exposure status and outcomes. Incomplete or unavailable address data, which is more common among certain sociodemographic groups, could bias results. However, only 1.5% of addresses were omitted due to low accuracy, suggestive of little potential bias. Changes in census tract measures over time and across residential histories, unmeasured in this study, could lead to exposure misclassification. We did not evaluate the potential effect modification of race and ethnicity. Future studies should evaluate potential effect modification by race and ethnicity to determine whether neighborhood disadvantage differentially affects outcomes across groups. Finally, concentrating solely on persistent poverty and low socioeconomic areas may underestimate the diverse, multifaceted barriers encountered by families enduring long‐term socioeconomic hardships [43, 44].

## 5 Conclusions

Our study confirms that chronic and contemporaneous neighborhood‐level socioeconomic hardships each independently contribute to significantly higher all‐cause and HCC‐specific mortality risks. These results reinforce the pressing need for comprehensive, multilevel efforts aimed at reducing the pervasive impact of structural disadvantage on liver cancer outcomes. Moving forward, prioritizing robust surveillance and intervention programs guided by integrated social and clinical data can help improve survival among those most vulnerable to HCC mortality and shrink inequities across diverse communities.

## Author Contributions

**Mohamed I. Elsaid:** conceptualization (lead), data curation (lead), formal analysis (lead), funding acquisition (lead), investigation (lead), methodology (lead), project administration (lead), resources (lead), software (lead), supervision (lead), validation (lead), visualization (lead), writing – original draft (lead), writing – review and editing (lead). **Vinod K. Rustgi:** writing – review and editing (equal). **Khalid Mumtaz:** writing – original draft (equal). **Chyke Doubeni:** writing – review and editing (equal). **Cecilia Dapino:** data curation (equal), formal analysis (equal), software (equal). **Electra D. Paskett:** writing – review and editing (equal). **Holli A. Loomans‐Kropp:** conceptualization (equal), writing – original draft (equal), writing – review and editing (equal). **Jesse J. Plascak:** writing – original draft (equal), writing – review and editing (equal). **Na Li:** writing – review and editing (equal). **Samilia Obeng‐Gyasi:** writing – review and editing (equal). **Yesung Kweon:** data curation (equal), formal analysis (equal), methodology (equal), software (equal).

## Funding

This work was supported by National Cancer Institute award 1L60CA305768‐01 and internal funding from The Ohio State University College of Medicine and The Ohio State University Comprehensive Cancer Center.

## Conflicts of Interest

Dr. Elsaid receives research funding from Genentech and AstraZeneca, all of which are unrelated to the current work. Dr. Paskett receives funding from AstraZeneca, Genentech, Pfizer, Merck Foundation, and Guardant Health and is a consultant for GSK and Merck, all of which are unrelated to the current work.

## Supporting information


**Data S1:** cam471721‐sup‐0001‐TableS1‐S3‐FigureS1‐S1@PP_SES_Supp_CM_R.docx.
**Table S1:** Sociodemographic, Clinical, and Treatment Characteristics of Patients with Hepatocellular Carcinoma by Residence in Low Socioeconomic Census Tract Status, The Surveillance, Epidemiology, and End Results (SEER) 2006–2020 (*n* = 50, 533).
**Figure S1:** Cumulative Risk of Mortality, Assessed by Exposure Of (1) Persistent Poverty And (2) Socioeconomic Status Census Tract. Populations Were Stratified by Residence in Persistent Poverty (Versus Not Residing In Persistent Poverty) For (A) All‐Cause And (B) HCC‐Specific Mortality And Residing In Low Socioeconomic Census Tracts (Versus Not Residing In Low Socioeconomic Census Tracts) For (C) All‐Cause And (D) HCC‐Specific Mortality.
**Table S3:** Associations Between Residing in (1) Persistently Impoverished Census Tract or (2) Low Socioeconomic Census Tract and All‐Cause and Hepatocellular Carcinoma Specific Mortality in Patients with Hepatocellular Carcinoma, The Surveillance, Epidemiology, and End Results (SEER), 2006–2020.
**Table S2:** Associations Between Residing in (1) Persistently Impoverished Census Tract or (2) Low Socioeconomic Census Tract and All‐Cause and Hepatocellular Carcinoma Specific Mortality in Patients with Hepatocellular Carcinoma, The Surveillance, Epidemiology, and End Results (SEER), 2006–2020.

## Data Availability

All data used in this study are publicly available from the National Institutes of Health and the National Cancer Institute. The corresponding author will provide the statistical codes upon request.
