# Supplementary material for: Impacts of Neighborhood Persistent Poverty and Socioeconomic Status on Hepatocellular Carcinoma Outcomes: A Large Population‐Based Cohort Study
Source: Cancer Med. 2026 Mar 17;15(3):e71721. doi: 10.1002/cam4.71721 (PMC13093718; doi:10.1002/cam4.71721)
Supplement: Supplementary file 1 — Data S1: cam471721‐sup‐0001‐TableS1‐S3‐FigureS1‐S1@PP_SES_Supp_CM_R.docx. Table S1: Sociodemographic, Clinical, and Treatment Characteristics of Patients with Hepatocellular Carcinoma by Residence in Low Socioeconomic Census Tract Status, The Surveillance, Epidemiology, and End Results (SEER) 2006–2020 (n = 50, 533). Figure S1: Cumulative Risk of Mortality, Assessed by Exposure Of (1) Persistent Poverty And (2) Socioeconomic Status Census Tract. Populations Were Stratified by Residence in Persistent Poverty (Versus Not Residing In Persistent Poverty) For (A) All‐Cause And (B) HCC‐Specific Mortality And Residing In Low Socioeconomic Census Tracts (Versus Not Residing In Low Socioeconomic Census Tracts) For (C) All‐Cause And (D) HCC‐Specific Mortality. Table S3: Associations Between Residing in (1) Persistently Impoverished Census Tract or (2) Low Socioeconomic Census Tract and All‐Cause and Hepatocellular Carcinoma Specific Mortality in Patients with Hepatocellular Carcinoma, The Surveillance, Epidemiology, and End Results (SEER), 2006–2020. Table S2: Associations Between Residing in (1) Persistently Impoverished Census Tract or (2) Low Socioeconomic Census Tract and All‐Cause and Hepatocellular Carcinoma Specific Mortality in Patients with Hepatocellular Carcinoma, The Surveillance, Epidemiology, and End Results (SEER), 2006–2020. [file CAM4-15-e71721-s001.docx]

**SUPPLEMENTARY Online Content**

**eMethods 1.** Exposure Definitions

**eMethods 2.** Statistical Analysis

**Supplementary Table S1.** Sociodemographic, Clinical, and Treatment Characteristics of Patients with Hepatocellular Carcinoma by Residence in Low Socioeconomic Census Tract Status, The Surveillance, Epidemiology, and End Results (SEER) 2006–2020 (n=50,533)

**Supplementary Figure S1**. Cumulative Risk of Mortality, Assessed by Exposure Of (1) Persistent Poverty And (2) Socioeconomic Status Census Tract. Populations Were Stratified by Residence in Persistent Poverty (Versus Not Residing In Persistent Poverty) For (A) All-Cause And (B) HCC-Specific Mortality And Residing In Low Socioeconomic Census Tracts (Versus Not Residing In Low Socioeconomic Census Tracts) For (C) All-Cause And (D) HCC-Specific Mortality.

**Supplementary Table S3.** Associations Between Residing in 1) Persistently Impoverished Census Tract or 2) Low Socioeconomic Census Tract and All-Cause and Hepatocellular Carcinoma Specific Mortality in Patients with Hepatocellular Carcinoma, The Surveillance, Epidemiology, and End Results (SEER), 2006–2020

**eMethods 1.**

**Exposure Definitions**

The study included two primary exposures: 1) residing in a persistently impoverished census tract and 2) residing in a low socioeconomic census tract.^1^ The persistent poverty variable identifies census tracts where at least 20% of the population has lived below the poverty level for approximately 30 years, using data from the 1990 and 2000 censuses and American Community Survey (**ACS**) 5-year estimates from 2007-2011 and 2015-2019. Developed by the **NCI** in collaboration with the US Department of Agriculture, this variable categorizes census tracts as either residing or not residing in a persistently impoverished census tract. The SES Index defined residence in a low SES census tract as follows. The SEER program constructed the SES Index scores based on census tract-level ACS 5-year estimates following a two-step process. First, composite SES scores were derived using factor analysis of seven key variables reflecting SES dimensions. These key variables included median household income, median house value, median rent, proportion below 150% of the poverty line, education index, percent working class, and percent unemployed.^2^ Second, census tracts were categorized into quintiles, with equal populations in each quintile across the US. Tumor cases were linked to SES quintiles based on diagnosis year and the corresponding ACS 5-year estimate. The first quintile, representing the lowest SES group, includes census tracts at or below the 20^th^ percentile. In contrast, the fifth quintile, representing the highest SES group, consists of those at or above the 80^th^ percentile. For our study, patients in the first SES quintile were considered to reside in a low socioeconomic census tract.

**eMethods 2.**

**Statistical Analysis**

We used Overlap Propensity Score Weighting (**OPSW**) to account for the impact of confounding resulting from the imbalances in baseline sociodemographic, clinical, and treatment variables between exposure groups.^3^ Overlap weighting is a propensity score method that attempts to imitate important features of randomized clinical trials, such as covariate balance and statistical precision. In simulation studies, overlap weighting showed improved covariate balance and statistical precision compared to other weighting methods, such as Inverse Probability of Treatment Weighting (**IPTW**).^3^ Additionally, overlap propensity score weighting has desirable statistical properties because it leads to an exact balance of the means and proportion of every covariate included in the logistic regression models used to estimate the propensity scores.

Propensity scores were estimated for cohorts one and two using two separate multivariable logistic regression models with 1) residing in a persistently impoverished census tract (cohort one) and 2) residing in a low socioeconomic census tract (cohort two) as the outcomes. Variables included in **Table 1** and **Supplementary Table S1** and their first-order interactions were considered covariates in the logistic models. We employed a forward effect selection approach, adding covariates and first-order interaction terms one at a time and retaining those significantly improved model fit (P < 0.05) or altered effect estimates by >10%. This strategy minimized overfitting and preserved all relevant confounding factors identified by our DAGs. Overlap weights were estimated separately for each cohort using the estimated propensity scores from each cohort. Overall and stratified characteristics were summarized using descriptive statistics, including means and Standard Deviations (**SD**) for continuous variables and frequencies and proportions for categorical variables. Differences between exposure groups were compared using Student's t-tests or Wilcoxon Rank Sum tests for continuous variables and χ^2^ or Fisher's exact tests for categorical variables. We used overlap-weighted standardized mean differences to compare sociodemographic, clinical, and treatment variables according to exposure status. A standardized difference of less than 0.1 indicated negligible differences between patient characteristics and exposure status.^4^

Cox proportional hazard models assume a constant hazard ratio over time and often report a single, weighted average effect estimate. This can obscure time-varying effects and complicate causal interpretations due to potential selection bias from conditioning on survival.^5^ We can flexibly account for time-varying hazards and interactions between exposure(s) and time by employing marginal structural models with parametric pooled logistic regression. This leads to a more accurate estimation of the causal effects of persistent poverty and census tract SES on survival in patients with HCC. Accordingly, we fitted marginal structural models using parametric pooled logistic regression, including an indicator for the exposure group, a flexible time-varying intercept (with linear and quadratic terms), and interaction terms between the exposure group and time. All marginal structural models were weighted using **OPSW.** The average 1, 5, 10, and 15-year absolute all-cause and HCC-specific mortality risks for each exposure group were estimated using the predicted values from the weighted marginal structural models. The resulting risk differences (**RDs**) and risk ratios (**RRs**) were then calculated. We used non-parametric bootstrapping with 1,000 replications to estimate 95% Confidence Intervals (**CIs**) for RDs and RRs.

We conducted secondary analyses using Kaplan-Meier and Cox proportional hazards regression to enhance the comparability of our findings with other studies. Incidence rates were estimated by dividing the outcome-specific deaths by each exposure group's total person-years of follow-up. We also calculated the absolute Incidence Rate Differences (**IRDs**) of all-cause and HCC-specific mortality for all outcomes by subtracting the incidence rates for the exposed and unexposed groups. **OPSW** Cox proportional hazards regression models were fitted to assess the relationships between the two exposures, all-cause and HCC-specific mortalities, separately. In a sensitivity analysis, we extended the study cohorts to include participants with AJCC stage IV HCC and those with distant or metastatic disease. We then repeated the OPSW Cox proportional hazards regression analyses on the extended cohorts. Two-sided tests were considered statistically significant, with a significance level of 0.05. All analyses were performed using SAS version 9.4. and R 4.2.0.

| **Supplementary Table S1.** Sociodemographic, Clinical, and Treatment Characteristics of Patients with Hepatocellular Carcinoma by Residence in Low Socioeconomic Census Tract Status, The Surveillance, Epidemiology, and End Results (SEER) 2006–2020 (n=50,533) | | | | | | | |
| --- | --- | --- | --- | --- | --- | --- | --- |
|  |  |  |  |  |  |  |  |
| **Patient Characteristics** | **Unweighted** | | |  | **Weighted*** | | |
|  | **Not Residing in Low Socioeconomic Census Tract^a^** | **Residing in Low Socioeconomic Census Tract^a^** | **Standardized Difference^†^** |  | **Not Residing in Low Socioeconomic Census Tract** | **Residing in Low Socioeconomic Census Tract** | **Standardized Difference^§^** |
|  |  |  |  |  |  |  |  |
|  | **n (%)** | **n (%)** |  |  | **%^‡^** | **%^‡^** |  |
| **No. of Patients** | 40670 (80.5) | 9863 (19.5) |  |  | 50.0 | 50.0 |  |
| **Demographics** |  |  |  |  |  |  |  |
| **Age,** Years |  |  |  |  |  |  |  |
| Mean (SD) | 64.1 (10.6) | 62.5 (9.9) | 0.1631 |  | 63.1 (3.5) | 62.9 (7.0) | 0.0251 |
| Median (25^th^, 75^th^) | 63.0 (57.0, 68.0) | 62.0 (56.0, 68.0) |  |  | 62.0 (57.0, 69.0) | 62.0 (56.0, 69.0) |  |
| **Age Group**, Years |  |  |  |  |  |  |  |
| 20 to 50 | 3054 (7.5) | 827 (8.4) | 0.0324 |  | 8.2 | 8.2 | 0^\|\|^ |
| 51 to 60 | 12440 (30.6) | 3548 (36.0) | 0.1145 |  | 34.2 | 34.2 |  |
| 61 to 70 | 14606 (35.9) | 3578 (36.3) | 0.0076 |  | 36.3 | 36.3 |  |
| 71+ | 10570 (26.0) | 1910 (19.4) | 0.1587 |  | 21.3 | 21.3 |  |
| **Sex** |  |  |  |  |  |  |  |
| Male | 30938 (76.1) | 7395 (75.0) | 0.0254 |  | 74.9 | 74.9 | 0^\|\|^ |
| Female | 9732 (23.9) | 2468 (25.0) |  |  | 25.1 | 25.1 |  |
| **Race-Ethnicity** |  |  |  |  |  |  |  |
| Non-Hispanic White | 20990 (51.6) | 3409 (34.6) | 0.3495 |  | 43.7 | 43.7 | 0^\|\|^ |
| Non-Hispanic Black | 3525 (8.7) | 2691 (27.3) | 0.4997 |  | 18.5 | 18.5 |  |
| Non-Hispanic American Indian/Alaska Native | 374 (0.9) | 97 (1.0) | 0.0066 |  | 1 | 1 |  |
| Non-Hispanic Asian or Pacific Islander | 7684 (18.9) | 836 (8.5) | 0.3066 |  | 10.6 | 10.6 |  |
| Hispanic | 7999 (19.7) | 2816 (28.6) | 0.2088 |  | 26.1 | 26.1 |  |
| Non-Hispanic Unknown Race | 98 (0.2) | 14 (0.1) | 0.0227 |  | 0.2 | 0.2 |  |
| **Marital Status at Diagnosis** |  |  |  |  |  |  |  |
| Married or Partner | 22135 (54.4) | 3911 (39.7) | 0.2993 |  | 44.8 | 44.8 | 0^\|\|^ |
| Separated or Divorced | 5372 (13.2) | 1763 (17.9) | 0.1291 |  | 17 | 17 |  |
| Single Never Married | 7762 (19.1) | 2890 (29.3) | 0.2403 |  | 24.6 | 24.6 |  |
| Widowed | 3660 (9.0) | 884 (9.0) | 0.0013 |  | 9.3 | 9.3 |  |
| Unknown | 1741 (4.3) | 415 (4.2) | 0.0036 |  | 4.3 | 4.3 |  |
| **Year of Diagnosis** |  |  |  |  |  |  |  |
| 2006 | 1845 (4.5) | 343 (3.5) | 0.0540 |  | 3.5 | 3.5 | 0^\|\|^ |
| 2007 | 1987 (4.9) | 414 (4.2) | 0.0331 |  | 4.2 | 4.2 |  |
| 2008 | 2121 (5.2) | 445 (4.5) | 0.0327 |  | 4.5 | 4.5 |  |
| 2009 | 2421 (6.0) | 572 (5.8) | 0.0065 |  | 5.7 | 5.7 |  |
| 2010 | 2522 (6.2) | 582 (5.9) | 0.0126 |  | 5.9 | 5.9 |  |
| 2011 | 2679 (6.6) | 659 (6.7) | 0.0038 |  | 6.7 | 6.7 |  |
| 2012 | 2863 (7.0) | 698 (7.1) | 0.0015 |  | 7 | 7 |  |
| 2013 | 2949 (7.3) | 837 (8.5) | 0.0459 |  | 8.3 | 8.3 |  |
| 2014 | 3229 (7.9) | 780 (7.9) | 0.0012 |  | 8 | 8 |  |
| 2015 | 3289 (8.1) | 879 (8.9) | 0.0296 |  | 8.8 | 8.8 |  |
| 2016 | 3066 (7.5) | 715 (7.3) | 0.0111 |  | 7.3 | 7.3 |  |
| 2017 | 2947 (7.3) | 727 (7.4) | 0.0048 |  | 7.7 | 7.7 |  |
| 2018 | 3041 (7.5) | 754 (7.6) | 0.0063 |  | 7.5 | 7.5 |  |
| 2019 | 3101 (7.6) | 784 (8.0) | 0.0121 |  | 8.1 | 8.1 |  |
| 2020 | 2610 (6.4) | 674 (6.8) | 0.0167 |  | 6.7 | 6.7 |  |
| **Persistent Poverty Census Tract** |  |  |  |  |  |  |  |
| Yes | 740 (1.8) | 5084 (51.6) | 1.3594 |  | 13.2 | 13.2 | 0^\|\|^ |
| No | 39930 (98.2) | 4779 (48.5) |  |  | 86.8 | 86.8 |  |
| **Census Urban-Area Categorization** |  |  |  |  |  |  |  |
| All Urban | 29792 (73.3) | 7323 (74.3) | 0.0226 |  | 70.7 | 70.7 | 0^\|\|^ |
| Mostly Urban | 7107 (17.5) | 1487 (15.1) | 0.0650 |  | 16.7 | 16.7 |  |
| Mostly Rural | 2238 (5.5) | 429 (4.4) | 0.0533 |  | 5.6 | 5.6 |  |
| All Rural | 1533 (3.8) | 624 (6.3) | 0.1170 |  | 7 | 7 |  |
| **Clinical and Treatment Characteristics** |  |  |  |  |  |  |  |
| **Histological Type** |  |  |  |  |  |  |  |
| Hepatocellular carcinoma**,** NOS | 40296 (99.1) | 9766 (99.0) | 0.0066 |  | 99 | 99 | 0^\|\|^ |
| Hepatocellular carcinoma, Fibrolamellar | 73 (0.2) | 18 (0.2) | 0.0007 |  | 0.2 | 0.2 |  |
| Hepatocellular carcinoma, Scirrhous | 38 (0.1) | 11 (0.1) | 0.0057 |  | 0.1 | 0.1 |  |
| Hepatocellular carcinoma, Spindle Cell Variant | 20 (0.1) | 2 (0.0) | 0.0155 |  | 0 | 0 |  |
| Hepatocellular carcinoma, Clear Cell Type | 233 (0.6) | 62 (0.6) | 0.0072 |  | 0.6 | 0.6 |  |
| Hepatocellular carcinoma, Pleomorphic Type | 10 (0.0) | 4 (0.0) | 0.0088 |  | 0 | 0 |  |
| **SEER Summary Stage** |  |  |  |  |  |  |  |
| Localized | 25981 (63.9) | 6098 (61.8) | 0.0425 |  | 62.1 | 62.1 | 0^\|\|^ |
| Regional | 14689 (36.1) | 3765 (38.2) |  |  | 37.9 | 37.9 |  |
| **AJCC Staging** |  |  |  |  |  |  |  |
| I | 19788 (48.7) | 4672 (47.4) | 0.0257 |  | 47.6 | 47.6 | 0^\|\|^ |
| II | 10202 (25.1) | 2363 (24.0) | 0.0262 |  | 24.4 | 24.4 |  |
| III | 10680 (26.3) | 2828 (28.7) | 0.0541 |  | 28 | 28 |  |
| **Pathologic Grade** |  |  |  |  |  |  |  |
| Grade 1 | 4310 (10.6) | 999 (10.1) | 0.0154 |  | 10.2 | 10.3 | 0.0050 |
| Grade 2 | 6356 (15.6) | 1466 (14.9) | 0.0213 |  | 14.6 | 15.3 | 0.0187 |
| Grade 3 | 2395 (5.9) | 586 (5.9) | 0.0022 |  | 5.6 | 5.9 | 0.0126 |
| Grade 4 | 194 (0.5) | 42 (0.4) | 0.0076 |  | 0.5 | 0.4 | 0.0159 |
| Unknown | 27415 (67.4) | 6770 (68.6) | 0.0264 |  | 69.1 | 68.1 | 0.0217 |
| **TNM-T** |  |  |  |  |  |  |  |
| T0/T1 | 19625 (48.3) | 4653 (47.2) | 0.0216 |  | 47.4 | 47.4 | 0^\|\|^ |
| T2 | 10157 (25.0) | 2362 (24.0) | 0.0239 |  | 24.4 | 24.4 |  |
| T3 | 7026 (17.3) | 1857 (18.8) | 0.0404 |  | 18 | 18 |  |
| T4 | 1352 (3.3) | 358 (3.6) | 0.0167 |  | 3.7 | 3.7 |  |
| Tx | 2510 (6.2) | 633 (6.4) | 0.0101 |  | 6.6 | 6.6 |  |
| **TNM-N** |  |  |  |  |  |  |  |
| N0 | 38960 (95.8) | 9449 (95.8) | 0.0004 |  | 95.6 | 95.6 | 0^\|\|^ |
| N1 | 1207 (3.0) | 303 (3.1) | 0.0061 |  | 3.2 | 3.2 |  |
| Nx | 503 (1.2) | 111 (1.1) | 0.0103 |  | 1.3 | 1.3 |  |
| **Surgery Status** |  |  |  |  |  |  |  |
| None | 27305 (67.1) | 7408 (75.1) | 0.0698 |  | 70.2 | 73.3 | 0.0698 |
| Local tumor destruction | 5949 (14.6) | 1145 (11.6) | 0.0317 |  | 13.6 | 12.6 | 0.0317 |
| Wedge or segmental resection | 2650 (6.5) | 456 (4.6) | 0.0529 |  | 5.8 | 4.6 | 0.0529 |
| Lobectomy | 1477 (3.6) | 258 (2.6) | 0.0194 |  | 3 | 2.7 | 0.0194 |
| Extended lobectomy | 386 (1.0) | 54 (0.6) | 0.0293 |  | 0.8 | 0.6 | 0.0293 |
| Hepatectomy | 2772 (6.8) | 510 (5.2) | 0.0203 |  | 6.2 | 5.8 | 0.0203 |
| Other Surgery | 131 (0.3) | 32 (0.3) | 0.0265 |  | 0.3 | 0.4 | 0.0265 |
| **Radiotherapy** |  |  |  |  |  |  |  |
| Yes | 4522 (11.1) | 1026 (10.4) | 0.0231 |  | 10.9 | 10.8 | 0.0030 |
| Refusal | 158 (0.4) | 56 (0.6) | 0.0260 |  | 0.4 | 0.6 | 0.0242 |
| No/Unknown¶ | 35990 (88.5) | 8781 (89.0) | 0.0170 |  | 88.7 | 88.6 | 0.0025 |
| **Chemotherapy** |  |  |  |  |  |  |  |
| Yes | 17207 (42.3) | 3887 (39.4) | 0.0590 |  | 41.5 | 39.8 | 0.0358 |
| No/Unknown | 23463 (57.7) | 5976 (60.6) |  |  | 58.5 | 60.2 |  |
| **Time From Diagnosis to Treatment, Months** |  |  |  |  |  |  |  |
| Mean (SD) | 2.3 (2.3) | 2.4 (2.4) | 0.0658 |  | 2.3 (0.8) | 2.4 (1.7) | 0.0984 |
| Median (25^th^, 75^th^) | 2 (1, 3) | 2 (1, 3) |  |  | 2 (1, 3) | 2 (1, 3) |  |
| **Time From Diagnosis to Treatment,** Months |  |  |  |  |  |  |  |
| 1 month or less | 12743 (31.3) | 2602 (26.4) | 0.1094 |  | 29.6 | 26.8 | 0.0611 |
| 2 months | 7331 (18.0) | 1444 (14.6) | 0.0917 |  | 17.2 | 15.1 | 0.0551 |
| 3+ months | 9384 (23.1) | 2222 (22.5) | 0.0130 |  | 22.8 | 23.3 | 0.0118 |
| Unknown | 11212 (27.6) | 3595 (36.5) | 0.1912 |  | 30.5 | 34.8 | 0.0914 |
| **AFP Pretreatment Interpretation** |  |  |  |  |  |  |  |
| Negative | 8800 (21.6) | 1855 (18.8) | 0.0705 |  | 20.2 | 20.2 | 0^\|\|^ |
| Positive | 17996 (44.3) | 4736 (48.0) | 0.0757 |  | 46.9 | 46.9 |  |
| Unknown** | 13874 (34.1) | 3272 (33.2) | 0.0199 |  | 32.9 | 32.9 |  |
| **Tumor Size,** mm |  |  |  |  |  |  |  |
| Mean (SD) | 54.3 (61.6) | 56.9 (62.2) | 0.0410 |  | 55.2 (20.3) | 56.0 (45.0) | 0.0224 |
| Median (25^th^, 75^th^) | 40 (25, 67) | 42 (26, 70) |  |  | 40 (25, 70) | 41 (26, 69) |  |
| **Tumor Size Group,** mm |  |  |  |  |  |  |  |
| < 50 | 23399 (57.5) | 5224 (53.0) | 0.0920 |  | 54.5 | 54.5 | 0^\|\|^ |
| ≥ 50 | 14688 (36.1) | 3836 (38.9) | 0.0574 |  | 37.7 | 37.7 |  |
| Unknown | 2583 (6.4) | 803 (8.1) | 0.0691 |  | 7.8 | 7.8 |  |
| **Total Number of in situ/malignant Tumors** |  |  |  |  |  |  |  |
| Mean (SD) | 1.1 (0.3) | 1.1 (0.2) | 0.0840 |  | 1.1 (0.1) | 1.1 (0.2) | 0.0185 |
| Median (25^th^, 75^th^) | 1.0 (1.0, 1.0) | 1.0 (1.0, 1.0) |  |  | 1.0 (1.0, 1.0) | 1.0 (1.0, 1.0) |  |
| **Total Number of in situ/malignant Tumors**, n (%) |  |  |  |  |  |  |  |
| 1 | 38008 (93.5) | 9400 (95.3) | 0.0804 |  | 94.8 | 94.8 | 0^\|\|^ |
| 2+ | 2662 (6.6) | 463 (4.7) |  |  | 5.2 | 5.2 |  |
| ^a^ Low socioeconomic status (SES) census tract was defined as residing in the lowest SES Index quintile | | | | | | | |
| * Using overlap weighting. The weighting aims to construct a pseudo-sample in which residing in low socioeconomic census tract status is independent of the baseline demographics and cancer characteristics influencing the likelihood of residing in a persistent poverty census tract | | | | | | | |
| † Absolute difference in means or proportions divided by pooled standard deviation. The imbalance between residing and not residing in a low socioeconomic census tract is defined as an absolute value greater than 0.10; smaller values indicate better balance. | | | | | | | |
| ‡ Overlap weighted proportions | | | | | | | |
| § Overlap-weighted standardized differences. All patient baseline demographics and cancer characteristics were used to estimate the weights. | | | | | | | |
| \|\| Overlapping weighting resulted in an exact balance between residing and not residing in a low socioeconomic census tract group for this variable | | | | | | | |
| ¶ Including recommended and unknown if administered | | | | | | | |
| ** Including test ordered, results not in the chart, information not collected, not documented, not assessed, or unknown if assessed | | | | | | | |
| SD, standard deviation, SES; socioeconomic status; NOS, not otherwise specified | | | | | | | |


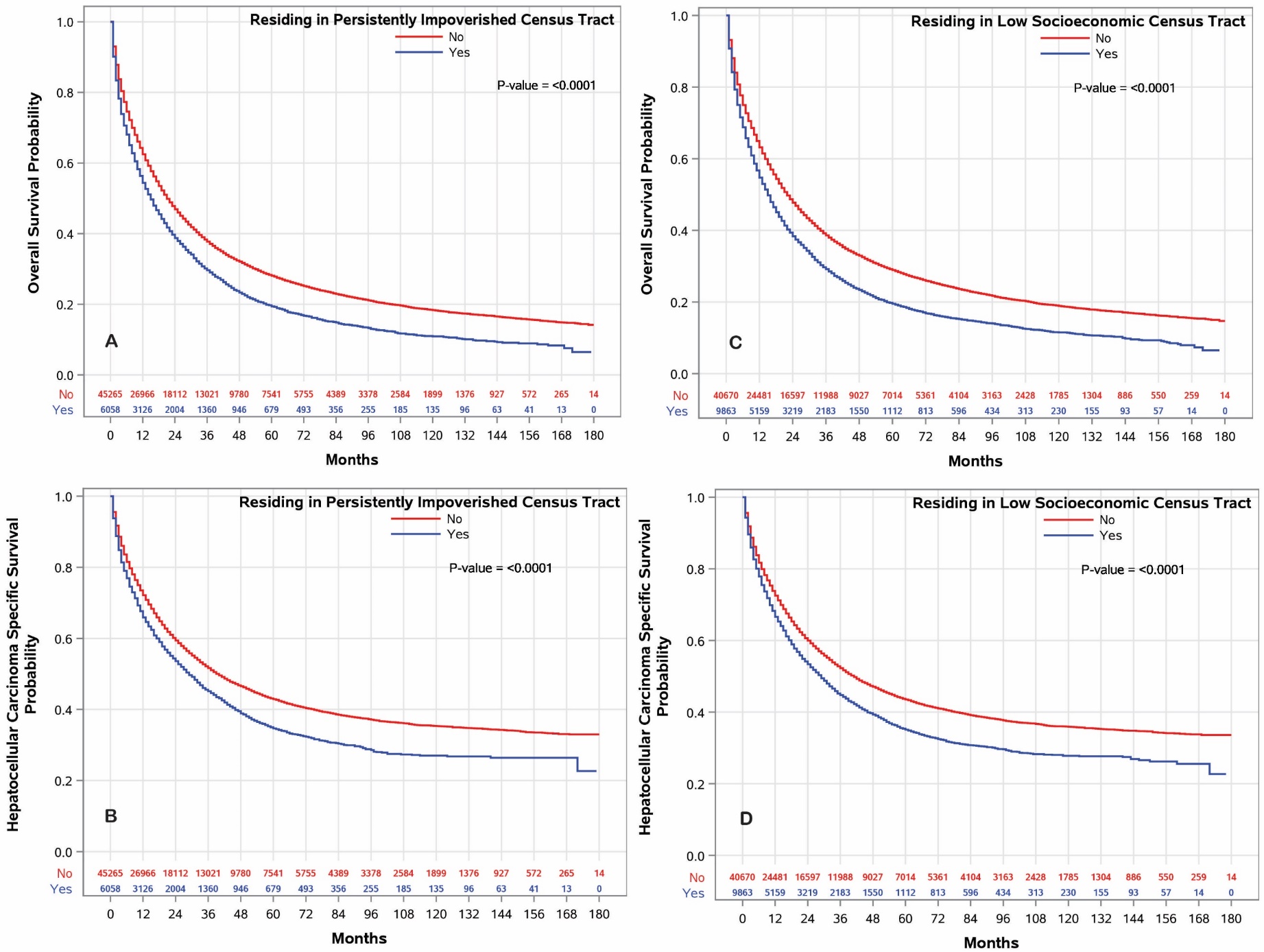


**Supplementary Figure S1**. Cumulative Risk of Mortality, Assessed by Exposure Of (1) Persistent Poverty And (2) Socioeconomic Status Census Tract. Populations Were Stratified by Residence in Persistent Poverty (Versus Not Residing In Persistent Poverty) For (A) All-Cause And (B) HCC-Specific Mortality And Residing In Low Socioeconomic Census Tracts (Versus Not Residing In Low Socioeconomic Census Tracts) For (C) All-Cause And (D) HCC-Specific Mortality.

| **Supplementary Table S2.** Associations Between Residing in 1) Persistently Impoverished Census Tract or 2) Low Socioeconomic Census Tract and All-Cause and Hepatocellular Carcinoma Specific Mortality in Patients with Hepatocellular Carcinoma, The Surveillance, Epidemiology, and End Results (SEER), 2006–2020 | | | | | | |
| --- | --- | --- | --- | --- | --- | --- |
|  |  |  |  |  |  |  |
| **Outcomes** | **Number of Participants** | **Number of Events** | **Person-Years** | **Incidence Rate Per 100 Person-Years** | **Absolute Incidence Rate Difference Per 100 Person-Years** | **Adjusted^‡^ Hazard Ratio** |
|  |  |  |  |  |  |  |
|  |  |  |  | **(95% CI)** | **(95% CI)** | **HR (95% CI)** |
| **All-Cause Mortality** | | | | | | |
| **Residing in Persistently Impoverished Census Tract** |  |  |  |  |  |  |
| Yes | 6,058 | 4,589 | 12,142.4 | 37.8 (36.7 to 38.9) | 10.8 (9.7 to 12.0) | 1.19 (1.15 to 1.22) |
| No | 45,265 | 31,080 | 115,299.9 | 27.0 (26.7 to 27.3) |  | Ref. |
| **Residing in Low Socioeconomic Census Tract*** |  |  |  |  |  |  |
| Yes | 9,863 | 7,464 | 19,874.3 | 37.6 (36.7 to 38.4) | 11.4 (10.5 to 12.3) | 1.16 (1.13 to 1.20)^‡^ |
| No | 40,670 | 27,684 | 105,802.7 | 26.2 (25.9 to 26.5) |  | Ref. |
| **Hepatocellular Carcinoma Specific Mortality** | | | | | | |
| **Residing in Persistently Impoverished Census Tract** |  |  |  |  |  |  |
| Yes | 6,058 | 2,995 | 12,142.4 | 24.7 (23.8 to 25.6) | 6.7 (5.7 to 7.6) | 1.15 (1.11 to 1.20) |
| No | 45,265 | 20,744 | 115,299.9 | 18.0 (17.7 to 18.2) |  | Ref. |
| **Residing in Low Socioeconomic Census Tract*** |  |  |  |  |  |  |
| Yes | 9,863 | 4,840 | 19,874.3 | 24.4 (23.7 to 25.0) | 6.8 (6.1 to 7.4) | 1.11 (1.07 to 1.16)^‡^ |
| No | 40,670 | 18,560 | 105,802.7 | 17.5 (17.3 to 17.8) |  | Ref. |
| * Low socioeconomic status (SES) Census Tract was defined as residing in the lowest SES Index quintile | | | | | | |
| † Adjusted using overlapping weights estimated using sex, age, race-ethnicity, marital status at diagnosis, year of diagnosis, census urban-area categorization, hepatocellular carcinoma histological type, seer summary stage, AJCC staging, TNM-N, TNM-T, AFP pretreatment interpretation, tumor size, and total number of in situ/malignant tumors | | | | | | |
| ‡ Adjusted using overlapping weights estimated using sex, age, race-ethnicity, marital status at diagnosis, year of diagnosis, census urban-area categorization, hepatocellular carcinoma histological type, seer summary stage, AJCC staging, TNM-N, TNM-T, AFP pretreatment interpretation, tumor size, total number of in situ/malignant tumors, and residing persistently impoverished census tract | | | | | | |
| HR= Hazard Ratio, CI = confidence interval | | | | | | |

**References**

1. Bhattacharya M, Cronin KA, Farrigan TL, Kennedy AE, Yu M, Srinivasan S. Description of census-tract-level social determinants of health in cancer surveillance data. *J Natl Cancer Inst Monogr*. Aug 1 2024;2024(65):152-161. doi:10.1093/jncimonographs/lgae027

2. Yost K, Perkins C, Cohen R, Morris C, Wright W. Socioeconomic status and breast cancer incidence in California for different race/ethnic groups. *Cancer Causes Control*. Oct 2001;12(8):703-11. doi:10.1023/a:1011240019516

3. Li F, Morgan KL, Zaslavsky AM. Balancing covariates via propensity score weighting. *Journal of the American Statistical Association*. 2018;113(521):390-400.

4. Austin PC. An introduction to propensity score methods for reducing the effects of confounding in observational studies. *Multivariate behavioral research*. 2011;46(3):399-424.

5. Hernán MA. The hazards of hazard ratios. *Epidemiology*. Jan 2010;21(1):13-5. doi:10.1097/EDE.0b013e3181c1ea43
